# Effects of General Fatigue Induced by Exhaustive Exercise on Posture and Gait Stability of Healthy Young Men

**DOI:** 10.3390/bs11050072

**Published:** 2021-05-08

**Authors:** Marinella Coco, Donatella Di Corrado, Francesco Cirillo, Chiara Iacono, Vincenzo Perciavalle, Andrea Buscemi

**Affiliations:** 1 Department of Biomedical and Biotechnological Sciences, University of Catania, 95123 Catania, Italy; marinella.coco@gmail.com; 2 Motor Activity Research Center (CRAM), University of Catania, 95123 Catania, Italy; 3 Department of Sport Sciences, Kore University, Cittadella Universitaria, 94100 Enna, Italy; perciava@libero.it; 4 Sant’Angela Merici Foundation (Scientific Director), 96100 Siracusa, Italy; cirillofranco45@gmail.com; 5 Euro Mediterranean Rehabilitation Summer School (President), 96100 Siracusa, Italy; 6 Medical Ragusa Supports, 97100 Ragusa, Italy; chiaraiacono28@gmail.com; 7 Horus Social Cooperative, Department of Research, 97100 Ragusa, Italy; andreabuscemi@virgilio.it; 8 Italian Center Studies of Osteopathy, Department of Research, 95100 Catania, Italy

**Keywords:** posture, blood lactate, upright position, exhaustive exercise

## Abstract

Bipedal walking is a composite task requiring integration of many control circuitries in the brain and spinal cord. The present study was carried out to verify whether an increase in blood lactate, such as that associated with a high intensity exercise, is able to significantly modify the qualitative and/or quantitative aspects of human walking. Eighteen healthy physically male participants, aged between 20 and 24 years (*M* = 21.8, *SD* = 1.22), were recruited for the study. For this purpose, the experimental protocol included the measure of blood lactate levels with the aim of assessing possible relations between lactate blood values and different aspect of walking after an exhaustive exercise. An exhaustive exercise was associated with a strong increase of blood lactate levels and produced a significant worsening in the ability to maintain the bipodalic upright posture as well as the fluidity of walking. Our results suggest that exhausting bouts impose greater challenges on postural control.

## 1. Introduction

Balance is usually described as the ability to maintain an upright position in a precise spatial orientation or to recover equilibrium after external dynamic perturbations, and it is in relation with the inertial forces acting on the body and the inertial features of his segments [1]. Preserving postural control under static or dynamic conditions is indispensable for quotidian life activities. The central nervous system and the musculoskeletal system are the main physiologic and neurological processes that control this state of remaining unaltered, even in the existence of forces that would usually change the condition [2]. Balance takes different forms: static, dynamic, stable, and unstable [3]. The state of remaining unchanged depends on proprioceptive information resulting from three areas: the sole of the foot, the cervical spine, and the sacroiliac joint [4]. Input from the sensorimotor system is then combined with information from the eyes and inner ear to control postural balance. Therefore, the somatosensory, visual, and vestibular systems are the three main systems controlling the ability to maintain an upright position, leading to the selection and execution of context-specific motor reactions [5]. For instance, the usual aptitude to close eyes during stance without loss of postural equilibrium comes from the ability of the somatosensory and vestibular perceptions to contribute adequate afferent information despite the lack of visual information. The appropriate function of the vestibular system influences the quality of body balance control, involving also the motor control, spatial coordination and effects of various forces on the human body.

Incapacity to produce the needed or expected power for performing any activity is defined as fatigue, which can be referred to a subjective symptom of malaise to activity or to objectively impaired performance or the lack of energy and motivation (both physical and mental). Irrespective of cause, it has a major impact on day-to-day functioning and quality of life [6]. Several studies highlighted the negative effect of exhausting exercise on postural control in upright stance [7,8,9]. When an individual performed a maximal exercise, a fast decrease in muscle phosphocreatine has been observed, as well as an accumulation of metabolites like lactate [10,11], influencing the normal functioning of several brain structures [12,13], such as the primary motor cortex and the supplementary motor area [14,15] obviously involved in human walking [16,17,18]. As aforementioned, postural control describes the way our central nervous system regulates sensory information from other systems (visual, vestibular, and somatosensory) in order to produce adequate motor output to maintain a controlled balance. Intensive general exercise increases postural sway when the energy expenditure induced exceeds the lactate accumulation threshold, probably altering the effectiveness of sensory inputs and motor output of postural control.

The present study was to verify whether an increase in blood lactate, such as that associated with a short and high intensity exercise, is able to significantly modify the qualitative and/or quantitative aspects of human walking. Our working hypothesis was that the possible influences exerted by an exhaustive activity on bipedal walking are linked with high levels of blood lactate. For this purpose, the experimental protocol included the measure of blood lactate levels (before the exercise and at the end of the exercise) with the aim of assessing possible relations between blood lactate values and different aspect of walking after an exhaustive exercise. A series of studies carried out over from 2009 has proven the central origin of the effects induced by the increase in blood lactate resulting from both a maximal physical exercise and an I.V. infusion of a lactate solution [19,20,21,22].

## 2. Materials and Methods

### 2.1. Participants

In order to have a sample of participants as homogeneous as possible, 20 young male volunteers were recruited for the study using convenience sampling. Participants were included if (a) they were aged 18 years or older, (b) currently they did not report any medication intake or relevant health impairments (e.g., orthopedic injury, neurological deficit, or vestibular impairment), (c) currently they were not involved in any professional sport, which could influence balance abilities, and (d) could provide informed consent. Participants were excluded if did not meet aforementioned inclusion criteria. Overall, 18 healthy physically male participants, aged between 20 and 24 years (*M* = 21.8, *SD* = 1.22) participated in this study. Measurements were conducted individually in a quiet location near the university, under the supervision of two researchers. All participants had normal or corrected-to-normal vision and performed recreational physical activity between two and three times per week. Each participant signed a free written consent to participate after receiving a full explanation of the goals and the protocol of the study. This study was carried out in accordance with the recommendations of the Ethical Code of the University of Palermo and of the Code of Ethics approved by the General Assembly of the Italian Association of Psychology held on 27 March 2015.

### 2.2. Procedure

To avoid circadian or circannual influences on posture and/or walking, the experiments were conducted between 10.00 a.m. and 1.00 p.m., and between October and December 2019. The participants were barefoot throughout the experiments. Firstly, each participant was instructed to stand on the platform and remain motionless for 10 s with open eyes and for another 10 s with closed eyes.

The experimental protocol consisted of three steps.

First step: the participant had, without interruption, to walk the platform back and forth four times, moving at the speed of his choice.

Second step: after completing this first gait test, the participant had to perform the exhaustive exercise.

Third step: at the end of the exhaustive exercise he immediately had to repeat the gait test of the first step, trying to move at the same speed as the first time.

**Figure 1:**
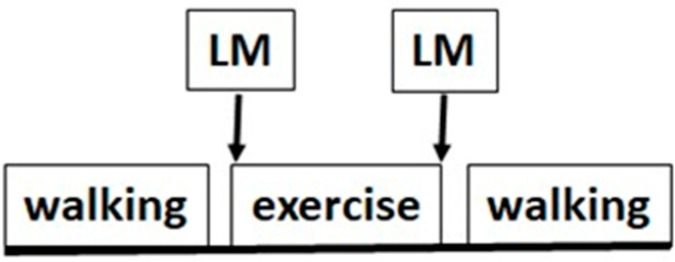
Experimental protocol. Note: LM: blood lactate measurement.

Blood lactate was measured before as well as at the end of the exhaustive exercise (Figure 1). The present experimental protocol is quite similar to those used in previous studies (e.g., [19,20,21,22]).

### 2.3. Measures

#### 2.3.1. Posture and Gait Assessment

Static and dynamic spatiotemporal parameters were obtained using the Body Analysis Kapture system, produced by Diagnostic Support (Greece), consisting of a 3-m pressure sensitive walkway (sampling rate: 100 Hz), integrated with a simultaneous video recording system with six cameras. The Body Analysis Kapture system was interfaced with an acquisition software version 2.0 Milletrix (Diagnostic Support, Greece). Concerning the posture, the acquisition system instantly evaluates the force components with respect to the position in the median–lateral direction (Xmean), anterior–posterior direction (Ymean), and vertical direction (Zmean), and the moment components about the x-, y-, z-axes. In particular, it was analyzed the area equivalent to 95% of the area designated by the center of pressure (CoP) trajectory (A95), because it has previously observed that this is the most efficient index of postural stability [23,24]. Concerning walking, main spatio-temporal data comprised the following values: velocity (km/h), cadence (steps/min), step length (cm), step time (s), single support phases (% gait cycle (%GC)), double support phases (%GC), and width between steps (cm).

#### 2.3.2. Exhaustive Exercise

The participants carried out a multistage incremental cycling test on a mechanically braked cycloergometer (Monark, Vansbro, Sweden), at a pedaling rate of 60 rpm. Each participant began with unloaded cycling for 3 min, and then, 30 W increased every 3 min, the load until the subject could no longer maintain the 60 rpm pedaling frequency [25].

#### 2.3.3. Blood Lactate

Blood lactate was measured before as well as at the end of the exercise, using a “Lactate Pro 2” portable lactate analyzer (Arkray Inc., Kyoto, Japan), as this portable device has a good reliability [26]. Blood samples were taken from the fingertip. The lactate analyzer produced the results of the blood lactate in 15 s after taking a blood sample.

### 2.4. Statistical Analysis

Data was collected and averaged; all descriptive statistics are reported as mean ± SD (level of significance: *p* ≤ 0.05). Differences in the mean of paired observations were analyzed with the non-parametric Wilcoxon matched-pairs signed rank test. All statistical analyses were processed with SPSS version 23 (SPSS Inc., Chicago, IL, USA).

## 3. Results

### 3.1. Participants

Anthropometric characteristics of participants are illustrated in Table 1.

### 3.2. Postural Control

**Figure 2:**
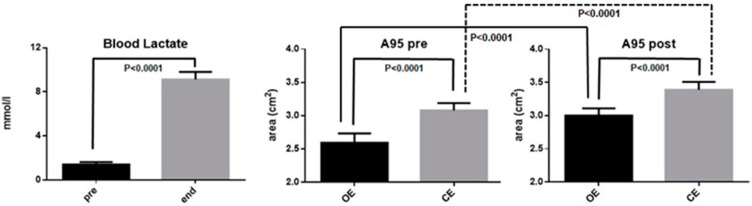
It can be seen, on the left, the mean value of blood lactate before and at the end of the exhaustive exercise. There are also shown the mean values of the area equivalent to 95% of the area designated by the CoP trajectory (A95), before and after the exercise. OE, open eyes; CE, closed eyes.

Figure 2 summarizes the obtained results. The exhaustive exercise increased in all the subjects the blood lactate levels from a mean value of 1.40 mmol/L (±0.23 SD) before the exercise (pre) to a mean value of 9.12 mmol/L (±0.69 SD) at the end of the exercise (end). Wilcoxon matched-pairs signed rank test showed that this difference was highly significant (*p* < 0.001).

Moreover, Figure 2 summarizes the results obtained when evaluating the A95 (in cm^2^). Comparing the data obtained from participants with open eyes (OE), with that when their eyes were closed (CE), it can be seen an increase of A95 with OE (2.59 cm^2^ ± 0.14 SD), with respect to the A95 mea value with CE (3.08 cm^2^ ± 0.09 SD). Wilcoxon matched-pairs signed rank test showed that this difference was highly significant (*p* < 0.001). In Figure 2 it can also be observed that, after exhaustive exercise, the subjects had a worsening of their postural control capacity, passing from a mean value of A95 value of 3.01 cm^2^ (±0.12 SD) with OE to a mean value of 3.39 cm^2^ (±0.11 SD) with CE. Wilcoxon matched-pairs signed rank test showed that this difference was highly significant (*p* < 0.001). The same statistical test detected statistically significant differences between the average values of the A95, both in OE and in CE, before and after the exercise.

### 3.3. Gate Control

Table 2 summarizes the obtained results. As can be seen, the exhaustive exercise caused a significant worsening of different aspects of gait.

The Wilcoxon matched-pairs signed rank test showed highly significant differences for all the studied variables, with the exception of the cadence that did not show statistically significant differences between before and after the exercise.

In summary, the obtained results revealed that, after an exhaustive exercise associated with a significant increase in blood lactate levels, the maintenance of the posture becomes less precise, both with and without visual input. Concerning the gait control, the results showed a worsening of almost all parameters taken into consideration. This is indicative of a gait that, while maintaining the cadence unchanged, revealed a reduction in the stride length, with consequent reduction in speed, associated with an increase of phases with both single and double support. Moreover, the exhaustive exercise, with the associated higher levels of blood lactate, determined an increase in the lateral distance between the two feet, an indication of the need of increasing the support polygon.

## 4. Discussion

Overall, the present study indicated that an exhaustive exercise is associated with a strong increase of blood lactate levels and produced a significant worsening in the ability to maintain the bipodalic upright posture as well as the fluidity of walking. In fact, the gait, although maintains unaffected the cadence, exhibited a reduction in the stride length, with consequent reduction in speed, associated with an increase of phases with both single and double support. Furthermore, the exhaustive exercise with the associated high levels of blood lactate induces an intensification of the need of increasing the support polygon.

During and succeeding a maximal exercise, there is a decrease in the ability of the muscles to produce force. Exhaustive exercises are frequently characterized by elevated energy demand; consequently, anaerobic energy metabolism plays a very important role. Thus, due to this mechanism, lactate production rises in the exercising muscles, as well as blood lactate concentration increases. These also causes a decline in the force production of muscle contractions, which results in a decrease in the muscle’s force to maintain postural control.

In line with results of the present study, it has been observed that postural stability decreases after an exhaustive working muscle [7,27,28]. Gauchard et al. [29] evaluated the effects of fatigue, with and without rehydration, on postural control in 10 healthy subjects. Results showed poor postural regulation, generally due to weaker muscular efficiency and lower proprioceptive or vestibular sensitivities. Zemková et al. [30] compared postural stability factors after maximum bouts on treadmill and cycling ergometer with different degrees of somatosensory stimulation, measuring heart rate and blood lactate. The results showed a more marked posture stability deterioration after maximum bout on treadmill in comparison to cycling.

Surenkok et al. [31] determined the effects of trunk-muscle fatigue and blood lactate elevation on static and dynamic balance, in sixteen healthy male university students. Significant differences in balance and lactate values were found after the fatigue protocol.

Santos et al. [32] evaluated the effects of judo combat on the athletes’ postural control before, during, and after a simulated match. Heart rate, blood lactate, and rating of perceived exertion were assessed. Authors concluded that one simulated judo match stimulates a significant metabolic response and balance is acutely degraded.

The present study was limited by the use of non-randomized trial. Moreover, only posture and gate of healthy young men were analyzed. Future studies should investigate the effects of exhaustive exercise on posture in women, to identify gender differences [33]. A second limitation was the lack of a correlation analysis to show how the levels or the change in the levels of lactate are related to the change in the spatio-temporal gait parameters. We, therefore, believe that this has not strongly affected our results, although it will have limited the statistical power.

Finally, we did not analyze the measurements of muscle phosphocreatine, as another biomarker. We recommend for future studies the analysis of these variables to have full gait analysis and to assess the fatigue effects on the different aspects of posture.

## 5. Conclusions

In conclusion, our hypotheses were partially confirmed. Changes in step width and stride duration coincided with a significant increase in blood lactate levels and appear to reflect compensations for impaired and decreased gait stability. These findings indicate that the modulations of gait may be an effect of the persistent decline of force generating capacity of muscle, imposing greater challenges on postural control. In addition, the balance deficits noted may specify an increased risk of injury with muscle fatigue not contributing to standing in an upright position.

## Figures and Tables

**Table 1:** Anthropometric characteristics of participants.

Participant	Age (years)	Height (cm)	Weight (kg)	BMI (kg/m^2^)	Shoes Size (US)
1	21	176	78	25.18	9
2	21	180	82	25.31	10
3	22	178	80	25.25	9.5
4	22	170	74	25.61	9
5	20	171	76	25.99	9
6	21	180	83	25.62	10
7	23	171	70	23.94	8.5
8	22	168	71	25.16	8.5
9	20	176	77	24.86	9
10	21	177	80	25.54	9.5
11	24	169	74	25.91	8.5
12	21	170	74	25.61	9
13	21	175	75	24.49	9.5
14	21	181	87	26.56	10
15	23	174	79	26.09	9
16	24	171	70	23.94	9
17	22	170	72	24.91	8.5
18	23	173	79	26.40	9
M	21.78	173.89	76.72	25.35	9.14
SD	1.22	4.14	4.71	0.74	0.51

**Table 2:** Spatio-temporal gait parameters of study participants (mean values ± SD) before (pre) and after (post) the exhaustive exercise. Results of Wilcoxon matched-pairs signed rank test between before and after the exercise are also shown.

Gait Variable	Velocity (km/h)	Cadence (steps/min)	Step Length (cm)	Step Time (s)	Single Support Phases (%GC)	Double Support Phases (%GC)	Width between Steps (cm)
Pre	3.54 (±0.32)	92.33 (±4.94)	58.76 (±4.98)	0.60 (±0.02)	35.56 (±1.94)	28.89 (±3.89)	11.06 (±1.35)
Post	3.03 (±0.29)	92.39 (±5.02)	54.76 (±4.94)	0.65 (±0.03)	33.89 (±1.75)	32.22 (±3.50)	15.11 (±2.13)
Pre-post Wilcoxon	<0.001	Non significant	<0.001	<0.001	<0.001	<0.001	<0.001
Cohen’s d	7.28	−0.04	−0.05	−7.02	4.34	−4.34	−3.00

## Data Availability

The data that support the findings of this study are available from the corresponding author (D.D.C.), upon reasonable request.
